# Effect of comorbidities and multimorbidity on bone mineral density in patients with osteoporosis

**DOI:** 10.1007/s11657-025-01604-6

**Published:** 2025-09-08

**Authors:** Luis Leal-Vega, María Begoña Coco-Martín, Adrián Martín-Gutiérrez, José Antonio Blázquez-Cabrera, Francisca Arranz-García, Amalia Navarro, María Jesús Moro, José Filgueira, Manuel Sosa-Henríquez, María Ángeles Vázquez, María José Montoya, Manuel Díaz-Curiel, José Manuel Olmos, José Luis Pérez-Castrillón, OSTEOMED Group

**Affiliations:** 1 Group of Applied Clinical Neurosciences and Advanced Data Analysis, Department of Medicine, Dermatology and Toxicology, University of Valladolid, Av. Ramón y Cajal, 7, 47005 Valladolid, Spain; 2 Internal Medicine Service, Albacete University Hospital Complex, Albacete, Spain; 3 Internal Medicine Service, San Carlos Clinical Hospital, Madrid, Spain; 4 Internal Medicine Service, Infanta Leonor University Hospital, Madrid, Spain; 5 Internal Medicine Service, Gregorio Marañón University Hospital, Madrid, Spain; 6 Internal Medicine Service, Insular University Hospital of Gran Canaria, Las Palmas, Spain; 7 Department of Medicine, University of Sevilla, Seville, Spain; 8 Internal Medicine Service, Jiménez Díaz Foundation University Hospital, Madrid, Spain; 9 Internal Medicine Service, Marqués de Valdecilla University Hospital, Cantabria, Spain; 10 Internal Medicine Service, Río Hortega University Hospital, Valladolid, Spain

**Keywords:** Osteoporosis, Comorbidity, Multimorbidity, Bone density, Dual-energy X-ray absorptiometry, Evidence-based practice

## Abstract

***Summary*:**

This retrospective cohort study analysed a total of 344 patients from the OSTEOMED registry with matched baseline and follow-up DXA data, finding that comorbidities such as nephrolithiasis, hypertension or coronary heart disease may influence the response to prescribed anti-osteoporotic treatment.

**Purpose:**

To determine: 1) comorbidities associated with reduced bone mineral density (BMD), T-score and Z-score at the lumbar spine (L1 to L4 vertebrae), femoral neck and total hip; and 2) the role of multimorbidity (≥ 2 comorbidities) in reduced BMD, T-score and Z-score at the lumbar spine, femoral neck and total hip.

**Methods:**

Retrospective cohort study analyzing patients [319 females (92.73%), 25 males (7.27%), age 62.13 ± 10.46 years] from the OSTEOMED registry with matched baseline and follow-up dual-energy X-ray absorptiometry (DXA) data. Patients' sex, age, body mass index (BMI), comorbidities and treatments were collected from their medical records after they had given written informed consent.

**Results:**

Considering a least significant change (LSC) of 4.2%, neither comorbidity nor multimorbidity was statistically significantly associated with a reduction in BMD in any of the bone regions studied. However, binary logistic regression analyses adjusted for sex, age, BMI and treatments showed that nephrolithiasis (*p* = 0.044) and coronary heart disease (*p* = 0.026) were statistically significantly associated with a reduction in total hip T-score and that hypertension (*p* = 0.049) and coronary heart disease (*p* = 0.01) were statistically significantly associated with a reduction in total hip Z-score.

**Conclusion:**

Despite comorbidity and multimorbidity, patients with osteoporosis are mostly well protected by anti-osteoporotic treatment in daily clinical practice. However, nephrolithiasis, hypertension, and coronary heart disease can influence the response to prescribed anti-osteoporotic treatment, especially at the total hip level.

## Introduction

Due to improved treatment of acute conditions and increased life expectancy, the leading causes of morbidity and mortality worldwide have shifted from infectious and parasitic diseases to non-communicable, chronic and degenerative diseases [1]. This phenomenon, known as the epidemiological transition, has important implications for the design and cost of healthcare services, as people with more long-term conditions have poorer health outcomes and make more frequent use of healthcare resources [2, 3].

In this sense, comorbidity is defined as the presence of at least 1 chronic disease together with an index disease and multimorbidity as the co-existence of ≥ 2 chronic diseases at the same time in an individual, although there is variability in how the latter should be measured [4]. Importantly, multimorbidity impacts on the functional ability of affected individuals and can be used as a prognostic indicator for hospitalisations, readmissions, length of stay, outcomes and survival [5–7].

Osteoporosis is a metabolic bone disease common in the elderly, a period of life in which other clinical entities are more likely to co-exist. It is therefore important to characterise the evolution of osteoporosis, as measured by changes in bone mineral density (BMD) or the occurrence of fractures, according to the comorbidities that patients present. Studies in this line of research have shown that the risk of fracture in patients with osteoporosis is increased by a wide range of conditions, such as hypertension, diabetes (especially type I), inflammatory bowel and joint diseases, breast and prostate cancer, and celiac disease [8–11], and that multimorbidity is associated with an increased risk of hip and fragility fractures [12, 13]. However, the number of studies evaluating the effects of comorbidity and multimorbidity on BMD in osteoporotic patients is scarce.

Given this gap in knowledge, and the fact that BMD is a central component of increased fracture risk, there is a critical need to profile patients at increased risk of BMD loss in order to design more effective management strategies. Therefore, this retrospective cohort study aims to determine the comorbidities independently associated with reduced BMD, T-score or Z-score at the lumbar spine (L1 to L4 vertebrae), femoral neck and total hip and the role of multimorbidity in reduced BMD, T-score or Z-score at the lumbar spine, femoral neck and total hip in a Spanish cohort of patients with osteoporosis followed up according to daily clinical practice.

## Methods

### Study design

Retrospective cohort study.

### Study population

Patients from the OSTEOMED registry, formed by those who attended internal medicine consultations in 23 Spanish hospitals for assessment and diagnosis of osteoporosis or the presence of fractures between 2012 and 2017. This registry includes data from 2,024 patients (1819 females (89.87%), 205 males (10.13%), age: 64.1 ± 12.1 years) and is promoted by the Working Group on Osteoporosis of the Spanish Society of Internal Medicine [14]. However, only those patients with matched baseline and follow-up DXA data from all bone regions considered were selected for the study.

Patients were mainly referred from primary care, other hospital services and other internal medicine practices. Patients diagnosed with osteoporosis according to the densitometric criteria established by the World Health Organization (WHO) (T-score < −2.5 at any location) or with typical fragility fractures regardless of BMD were included [15, 16]. Patients with neoplasia, life expectancy < 1 year or age > 90 years were excluded from the registry as their follow-up was considered unfeasible in the proposed manner. Patients were followed up according to standard clinical practice, with no additional diagnostic tests or therapeutic interventions during the study. All patients received an information sheet about the aim and risks of the study and signed a written informed consent before the collection of their clinical or personal data. This study was conducted in accordance with the Declaration of Helsinki [17] and was approved by the Clinical Research Ethics Committee of the Albacete University Hospital Complex (Act 02/11).

### Study variables

Patients were received in two visits (with at least 1 year between visits) in which their BMD, T-score and Z-score at the lumbar spine (L1 to L4 vertebrae), femoral neck and hip were determined by dual energy X-ray absorptiometry (DXA) and their sex, age, body mass index (BMI), comorbidities and prescribed non-osteoporotic and anti-osteoporotic treatments were collected from their medical records.

Comorbidities recorded included hypertension, dyslipidemia, diabetes mellitus, coronary heart disease, chronic kidney disease, chronic obstructive pulmonary disease, rheumatoid arthritis, breast cancer, prostate cancer, hypercalciuria, hyperthyroidism, hypothyroidism, celiac disease, Crohn’s disease and nephrolitiasis. Non-osteoporotic treatments recorded included radiotherapy, chemotherapy, tamoxifen, aromatase inhibitors, benzodiazepines, proton pump inhibitors, selective serotonin reuptake inhibitors, thiazide diuretics, statins, thyroid hormones, corticosteroids and gonadotropin-releasing hormone (GnRH) agonists. On the other hand, anti-osteoporotic treatments recorded included calcium, vitamin D, bisphosphonates, selective estrogen receptor modulators, strontium ranelate, denosumab, calcitonin, teriparatide and parathyroid hormone (PTH).

Numerical variables were created for BMD, T-score and Z-score determined at each visit and then categorical variables named “BMD variation”, “T-score variation” and “Z-score variation”, which took the value ‘1’ when there was a decrease of in their value between visits and “0” otherwise. For “BMD variation”, only a decrease ≥ 4.2% was considered, which refers to the so-called least significant change (LSC), resulting from multiplying 2.8 by the coefficient of variation of the technique used (here assumed to be 1.5%) [18]. Each comorbidity was a categorical variable that took the value ‘1’ when present and ‘0’ when absent. Another categorical variable called “multimorbidity” was created, which took the value ‘1’ when ≥ 2 comorbidities were present and ‘0’ when not. Each treatment was also a categorical variable that took the value ‘1’ when prescribed and ‘0’ when not."Polypharmacy"was considered when ≥ 5 drugs were prescribed [19].

### Statistical analysis

Binary logistic regression was used for the statistical analysis of the results of the study. Logistic regression is a predictive modelling technique that provides a predictive model to explain a dichotomous variable from independent variables. The function of the model is to predict the probability of belonging to a category or group (in our case the probability of reduced BMD, T-score and Z-score) according to the comorbidities and multimorbidity presented by the patients. Another measure calculated was the odds ratio (OR) associated with each comorbidity and multimorbidity, which reflects how many times the odds of a decrease are greater than the odds of an increase when present. Statistical analyses were performed with IBM® SPSS® Statistics v24.0 (IBM Corp., Armonk, NY) [20] and a P value < 0.005 was assumed for statistical significance.

## Results

A total of 344 patients [319 females (92.73%), 25 males (7.27%), age 62.13 ± 10.46 years] from the OSTEOMED registry with matched baseline and follow-up DXA data were analysed. The mean time between visits was 27.06 ± 13.37 months. The characteristics of the patients analysed are described in Table 1.

**Table 1:** Characteristics of patients (*n* = 344)

Sex	
Males	25 (7.27%)
Females	319 (92.73%)
Age	
< 65 y/o	207 (60.17%)
65–75 y/o	93 (27.04%)
> 75 y/o	44 (12.79%)
BMI (kg/m^2^)	25.86 ± 4.82
Comorbidities	
Hypertension	108 (31.4%)
Dyslipidemia	96 (27.91%)
Diabetes mellitus	24 (6.98%)
CHD	14 (4.07%)
CKD	4 (1.16%)
COPD	8 (2.33%)
Rheumatoid arthritis	2 (0.58%)
Breast cancer	37 (10.76%)
Prostate cancer	1 (0.29%)
Hypercalciuria	2 (0.58%)
Hyperthyroidism	6 (1.74%)
Hypothyroidism	35 (10.17%)
Celiac disease	2 (0.58%)
Crohn's disease	2 (0.58%)
Nephrolithiasis	12 (3.49%)
Multimorbidity	100 (29.07%)
Lumbar spine	
BMD variation (g/cm^2^)	0.01 ± 0.06
T-score variation	0.12 ± 0.64
Z-score variation	0.21 ± 0.57
Femoral neck	
BMD variation (g/cm^2^)	−0.01 ± 0.05
T-score variation	0.01 ± 0.57
Z-score variation	0.07 ± 0.65
Total hip	
BMD variation (g/cm^2^)	0.01 ± 0.07
T-score variation	0.09 ± 0.5
Z-score variation	0.17 ± 0.45
Non-osteoporotic treatments	
Radiotherapy	23 (6.69%)
Chemotherapy	22 (6.4%)
Tamoxifene	12 (3.49%)
Anastrozole	12 (3.49%)
Letrozole	5 (1.45%)
Exemestane	5 (1.45%)
GnRH agonists	1 (0.29%)
Oral corticosteroids	15 (4.36%)
Inhaled corticosteroids	9 (2.62%)
Proton pump inhibitors	49 (14.24%)
SSRIs	24 (6.98%)
Benzodiazepines	29 (8.43%)
Thyroid hormones	35 (10.17%)
Thiazide diuretics	11 (3.2%)
Statins	68 (19.77%)
Anti-osteoporotic treatments	
Calcium	244 (70.93%)
Vitamin D	272 (79.07%)
Alendronate	27 (7.85%)
Risedronate	56 (16.28%)
Ibandronate	10 (2.91%)
Zoledronate	3 (0.87%)
Raloxifene	4 (1.16%)
Bazedoxifene	5 (1.45%)
Strontium ranelate	7 (2.03%)
Denosumab	38 (11.05%)
Calcitonin	1 (0.29%)
Teriparatide	26 (7.56%)
Parathyroid hormone	7 (2.03%)
Polypharmacy	50 (14.53%)

*BMD *bone mineral density, *BMI *body mass index, *CHD *coronary heart disease, *CKD *chronic kidney disease, *COPD *chronic obstructive pulmonary disease, *GnRH *gonadotropin-releasing hormone, *SSRIs *selective serotonin reuptake inhibitors, *y/o *years old

### Bone mineral density

At follow-up, the BMD of the lumbar spine, femoral neck and total hip of the patients experienced a change of 0.12 ± 0.64, 0.01 ± 0.57 and 0.09 ± 0.5 g/cm^2^, respectively. A total of 256 (74.42%) patients experienced a reduction in BMD in any of the bone regions studied. A decrease in BMD at the lumbar spine, femoral neck and hip was observed in 140 (40.7%), 181 (52.62%) and 133 (38.66%) patients, respectively, while a decrease in BMD ≥ 4.2% at the lumbar spine, femoral neck and hip was observed in 64 (18.6%), 101 (29.36%) and 67 (19.48%) patients, respectively. After adjusting the model for sex, age, BMI and treatments, binary logistic regression analysis showed that neither comorbidity nor morbidity was statistically significantly associated with a decrease in BMD ≥ 4.2% in any of the bone regions studied.

## T-score

At follow-up, patients'lumbar spine, femoral neck and total hip T-scores experienced a change of 0.12 ± 0.64, 0.01 ± 0.57 and 0.09 ± 0.50, respectively. A total of 236 (68.6%) patients experienced a reduction in T-score in any of the bone regions studied. A decrease in T-score at the lumbar spine, femoral neck and total hip was observed in 119 (34.6%), 148 (43.02%) and 106 (30.81%) patients, respectively. After adjusting the model for sex, age, BMI and treatments, binary logistic regression analysis showed that nephrolithiasis (*p* = 0.044) and coronary heart disease (*p* = 0.026) were statistically significantly associated with a reduction in total hip T-score. The effect of comorbidities and multimorbidity on the reduction of total hip T-score can be found in Table 2.

**Table 2:** Effects of comorbidities and multimorbidity on total hip T-score reduction

	OR	95% CI	*p*-value
Hypertension	1.809	0.699–4.679	0.222
Dyslipidemia	0.370	0.109–1.255	0.111
Diabetes mellitus	3.756	0.761–18.545	0.104
CHD	49.911	1.608–1549.207	0.026*
CKD	n/a	n/a	n/a
COPD	0.308	0.008–11.707	0.526
Rheumatoid arthritis	5.588	0.111–281.745	0.39
Breast cancer	0.094	0.002–5.403	0.253
Prostate cancer	n/a	n/a	n/a
Hypercalciuria	n/a	n/a	n/a
Hyperthyroidism	4.107	0.226–74.494	0.339
Hypothyroidism	1.124	0.244–5.181	0.881
Celiac disease	n/a	n/a	n/a
Crohn's disease	n/a	n/a	n/a
Nephrolithiasis	11.428	1.073–121.759	0.044*
Multimorbidity	5.096	0.162–160.666	0.355

*95% CI *95% confidence interval, *CHD *coronary heart disease, *CKD *chronic kidney disease, *COPD *chronic obstructive pulmonary disease, *n/a *not available, *OR *odds ratio

*p-value < 0.05

### Z-score

At follow-up, patients'lumbar spine, femoral neck and total hip Z-scores experienced a change of 0.21 ± 0.57, 0.07 ± 0.65 and 0.17 ± 0.45, respectively. A total of 190 (55.23%) patients experienced a reduction in Z-score in any of the bone regions studied. A decrease in Z-score at the lumbar spine, femoral neck and total hip was observed in 90 (26.16%), 108 (31.4%) and 82 (23.84%) patients, respectively. After adjusting the model for sex, age, BMI and treatments, binary logistic regression analysis showed that hypertension (*p* = 0.049) and CHD (*p* = 0.01) were statistically significantly associated with a reduction in total hip Z-score. The effect of comorbidities and multimorbidity on the reduction of total hip Z-score can be found in Table 3.

**Table 3:** Effects of comorbidities and multimorbidity on total hip Z-score reduction

	OR	95% CI	*p*-value
Hypertension	2.827	1.007–7.94	0.049*
Dyslipidemia	0.213	0.43–1.06	0.059
Diabetes mellitus	4.391	0.739–26.087	0.104
CHD	103.38	2.987–3578.341	0.01*
CKD	n/a	n/a	n/a
COPD	2.383	0.95–59.488	0.597
Rheumatoid arthritis	4.222	0.087–205.372	0.467
Breast cancer	2.733	0.061–123.255	0.605
Prostate cancer	n/a	n/a	n/a
Hypercalciuria	n/a	n/a	n/a
Hyperthyroidism	5.198	0.178–151.722	0.338
Hypothyroidism	0.596	0.101–3.502	0.567
Celiac disease	n/a	n/a	n/a
Crohn's disease	n/a	n/a	n/a
Nephrolithiasis	6.303	0.793–50.084	0.082
Multimorbidity	1.204	0.046–31.505	0.911

*95% CI *95% confidence interval, *CHD *coronary heart disease, *CKD *chronic kidney disease, *COPD *chronic obstructive pulmonary disease, *n/a *not available, *OR *odds ratio

*p-value < 0.05

## Discussion

The relationship between comorbidity and poorer health outcomes in patients diagnosed with osteoporosis has been a controversial topic in the scientific literature. For example, Lix et al. [21] investigated the Charlson [22] and Elixhauser [23] indices for predicting mortality, hospitalisation and fractures in two cohorts of osteoporotic patients and found that only the Elixhauser index produced a statistically significant improvement in fracture prediction accuracy over the base set of explanatory variables, although the magnitude of change in discrimination was small. Likewise, in the FREEDOM trial [24], no statistically significant correlations were found between the Shanga [25] and Wolfe [26] indices and the number of vertebral fractures, although a modest significant correlation was observed with the number of fragility fractures prior to baseline. The GLOW study [27] did observe that the risk of fracture increased with various comorbidities such as Parkinson's disease, multiple sclerosis, and heart and lung disease, although at 3-year follow-up no increased risk of fracture was associated with these comorbidities [28]. In contrast, other studies do support that the risk of fracture in patients with osteoporosis is increased by the presence of various comorbidities [8–11] and multimorbidity [12, 13].

Previous research in a larger number of patients from this cohort showed that, in general, sex, age and number of comorbidities did not influence the response to anti-osteoporotic treatment, considered as incident fragility fractures after a minimum follow-up of 1 year [29]. Similarly, in this study we found no evidence that comorbidities or multimorbidity had a negative impact on patients'BMD when an LSC of 4.2% was taken into account. Nevertheless, we did observe that nephrolithiasis (*p* = 0.044) and coronary heart disease (*p* = 0.026) were statistically significantly associated with a reduction in total hip T-score, and that hypertension (*p* = 0.049) and coronary heart disease (*p* = 0.01) were statistically significantly associated with a reduction in total hip Z-score.

Nephrolithiasis, characterized by the formation of kidney stones, may be associated with a reduction in BMD through interconnected physiological mechanisms involving calcium homeostasis, renal function, and metabolic dysregulation. Hypercalciuria, a hallmark of calcium-based nephrolithiasis, can lead to an increase in parathyroid hormone to maintain calcaemia, often resulting in increased bone resorption, which depletes skeletal calcium stores and reduces BMD, especially in weight-bearing areas such as the hip. In this regard, studies have shown that people with nephrolithiasis tend to have lower BMD compared to controls [30, 31] and that men with a history of kidney stones have lower femoral neck BMD than men without a history of kidney stones after adjusting for confounding factors such as age or BMI [32], which is consistent with our findings.

On the other hand, hypertension promotes systemic inflammation and oxidative stress, which increases osteoclast activity and suppresses osteoblast activity, leading to lower BMD. Impaired vascular perfusion due to hypertension-related microvascular damage further compromises bone remodeling, especially in weight-bearing areas such as the hip. In addition, hypertension-related renal dysfunction may disrupt calcium and vitamin D homeostasis, exacerbating bone loss. Despite some conflicting evidence [33], our results support the conclusions of previous studies that have demonstrated a close relationship between hypertension and reduced BMD [34–38].

Finally, the relationship between coronary heart disease and reduced BMD could be due to numerous reasons. First, chronic inflammation associated with coronary heart disease, driven by elevated cytokines such as interleukin-6 (IL-6) and tumor necrosis factor-alpha (TNF-α), accelerates bone resorption by activating osteoclasts. Second, impaired cardiac function often leads to reduced physical activity, which decreases the mechanical load on bones, especially in weight-bearing areas such as the hip, resulting in lower BMD. Third, common risk factors such as advanced age or smoking exacerbate both coronary heart disease and bone density loss. Added to this are systemic hypoperfusion, which impairs bone remodeling, and hormonal imbalances such as estrogen or testosterone deficiency, which promote bone resorption. In this regard, our study aligns with previous studies that have found that patients with coronary heart disease have lower BMD in the femoral neck [39, 40] and a higher risk of subsequent hip fracture [41–43].

Among the strengths of this study are the sample size included and the robustness of the statistical methodology used, while the main limitations to be taken into account are the relatively short duration of follow-up, the lack of subjects without osteoporosis in the analysis, and the absence of different ethnicities in the study population and different comorbidities in the study variables. These conditions have been shown to increase the risk of fracture in osteoporotic patients [44, 45], but this may not be due to a reduction in BMD, but to an increased risk of falls due to disease symptoms or as an adverse effect of medications. Other comorbidities included in this study, such as chronic kidney disease, hypercalciuria, prostate cancer, Chron's disease and celiac disease could not be correlated with reduced BMD, T-scores and Z-scores in the statistical analysis. Future studies that include healthy subjects, more comorbidities, different ethnic groups, and longer follow-up could help validate the results obtained and fill existing knowledge gaps.

## Conclusion

Patients with osteoporosis are mostly well protected by anti-osteoporotic treatment in daily clinical practice. However, nephrolithiasis, hypertension, and coronary heart disease can influence the response to prescribed anti-osteoporotic treatment, especially at the total hip level.

## Data Availability

Data will be shared upon reasonable request to the corresponding author.
